# Tightly-Coupled Plant-Soil Nitrogen Cycling: Comparison of Organic Farms across an Agricultural Landscape

**DOI:** 10.1371/journal.pone.0131888

**Published:** 2015-06-29

**Authors:** Timothy M. Bowles, Allan D. Hollander, Kerri Steenwerth, Louise E. Jackson

**Affiliations:** 1 Department of Land, Air and Water Resources, University of California Davis, Davis, California, United States of America; 2 Information Center for the Environment, Department of Environmental Science and Policy, University of California, Davis, Davis, California, United States of America; 3 Crops Pathology and Genetics Research Unit, USDA/ARS, Davis, California, United States of America; North Carolina State University, UNITED STATES

## Abstract

How farming systems supply sufficient nitrogen (N) for high yields but with reduced N losses is a central challenge for reducing the tradeoffs often associated with N cycling in agriculture. Variability in soil organic matter and management of organic farms across an agricultural landscape may yield insights for improving N cycling and for evaluating novel indicators of N availability. We assessed yields, plant-soil N cycling, and root expression of N metabolism genes across a representative set of organic fields growing Roma-type tomatoes (*Solanum lycopersicum* L.) in an intensively-managed agricultural landscape in California, USA. The fields spanned a three-fold range of soil carbon (C) and N but had similar soil types, texture, and pH. Organic tomato yields ranged from 22.9 to 120.1 Mg ha^-1^ with a mean similar to the county average (86.1 Mg ha^-1^), which included mostly conventionally-grown tomatoes. Substantial variability in soil inorganic N concentrations, tomato N, and root gene expression indicated a range of possible tradeoffs between yields and potential for N losses across the fields. Fields showing evidence of tightly-coupled plant-soil N cycling, a desirable scenario in which high crop yields are supported by adequate N availability but low potential for N loss, had the highest total and labile soil C and N and received organic matter inputs with a range of N availability. In these fields, elevated expression of a key gene involved in root N assimilation, cytosolic glutamine synthetase GS1, confirmed that plant N assimilation was high even when inorganic N pools were low. Thus tightly-coupled N cycling occurred on several working organic farms. Novel combinations of N cycling indicators (i.e. inorganic N along with soil microbial activity and root gene expression for N assimilation) would support adaptive management for improved N cycling on organic as well as conventional farms, especially when plant-soil N cycling is rapid.

## Introduction

Reducing tradeoffs among ecosystem services related to nitrogen (N) cycling in agriculture is a global challenge [1]. One promising strategy to reduce such tradeoffs relies on a stronger role for biological processes to support high yields [2], such as practiced in organic agriculture, rather than non-renewable inputs like synthetic N fertilizer. Ideally soil microbial processing of organic matter releases sufficient N synchronously with crop N demand to lead to high productivity but with minimal N losses via leaching and denitrification (i.e. tightly-coupled N cycling) [3], thereby reducing tradeoffs among yields, water quality, and potentially greenhouse gas emissions [4–8].

The complex plant and microbial ecological processes related to N cycling are strongly influenced by factors that vary across agricultural landscapes and even across fields within an individual farm, especially soil physicochemical and biological factors and nutrient management [9–11]. Most research geared toward improving N cycling takes place at research stations with fixed factors and limited variation in soil characteristics [12], so little is known about how effective N cycling is achieved on working farms or about innovations that farmers use. Previous landscape-scale assessments of organic farms have focused on explaining yield variability, crop quality, or comparing management with conventional farms [11,13–15]. Understanding the plant-soil-microbe interactions that underpin N availability, potential for N loss, and yields across working organic farms would help reveal how to simultaneously achieve high provisioning (yields) and regulating (low potential for N loss) ecosystem services.

Adaptive management that implicitly recognizes variability and uncertainty among fields is a more viable approach for achieving tightly-coupled N cycling as compared to a one-size-fits-all prescription for management of soils and organic matter inputs [16,17]. But the lack of reliable indicators of N availability for organic systems limits the ability of growers to learn from and adapt management practices, and of researchers to accurately assess ecosystem services in organic systems [18]. For instance, the pre-sidedress soil nitrate (NO_3_^-^) test is used widely in conventional systems to indicate plant available N just before the exponential growth phase of the crop [19,20]. However, low soil NO_3_^-^ pools can occur even when N availability is high if soil NO_3_^-^ turns over rapidly, such as when high input (e.g. soil N mineralization) and high output (e.g. plant N uptake or microbial N immobilization) fluxes occur simultaneously [21]. The higher soil carbon (C) availability often resulting from organic management [22,23] can increase both microbial N demand and gross soil N transformation rates [10,24], thereby increasing plant-soil-microbe soil N cycling and turnover of inorganic N. Thus, new indicators of N availability are needed that take into account active C and N processes in organic systems [19]. Good candidates are labile soil organic matter (SOM) fractions [23,25], which will benefit from more on-farm validation and standardization.

Expression levels of genes involved in root N uptake and assimilation may also indirectly indicate plant available N in soil and provide a complement to biogeochemical indicators of N availability, especially when soil NO_3_^-^ turnover is high. Plant N uptake and assimilation systems respond to wide variation in external N availability and internal N metabolites that reflect plant N status [26–28] through regulatory mechanisms that optimize capture of limiting nutrients [29]. Recent work has expanded knowledge of plant root transcriptional responses to N availability from laboratory-based systems (e.g. hydroponics) into natural soil conditions, thus providing a basis for selecting candidate genes as indicators of soil N processes [30,31]. These genes include high-affinity transporters of NH_4_^+^ and NO_3_^-^; nitrite reductase, responsible for reduction of NO_3_^-^ to nitrite; and glutamine synthetase and glutamate synthase, which are involved in NH_4_^+^ assimilation into amino acids [32–34]. Analyzing expression of these genes in roots may provide a “plant’s eye view” of soil N availability, and show how root N assimilation is high even when soil inorganic N pools are low, i.e. in situations of tightly-coupled and rapid N cycling.

**Fig 1:**
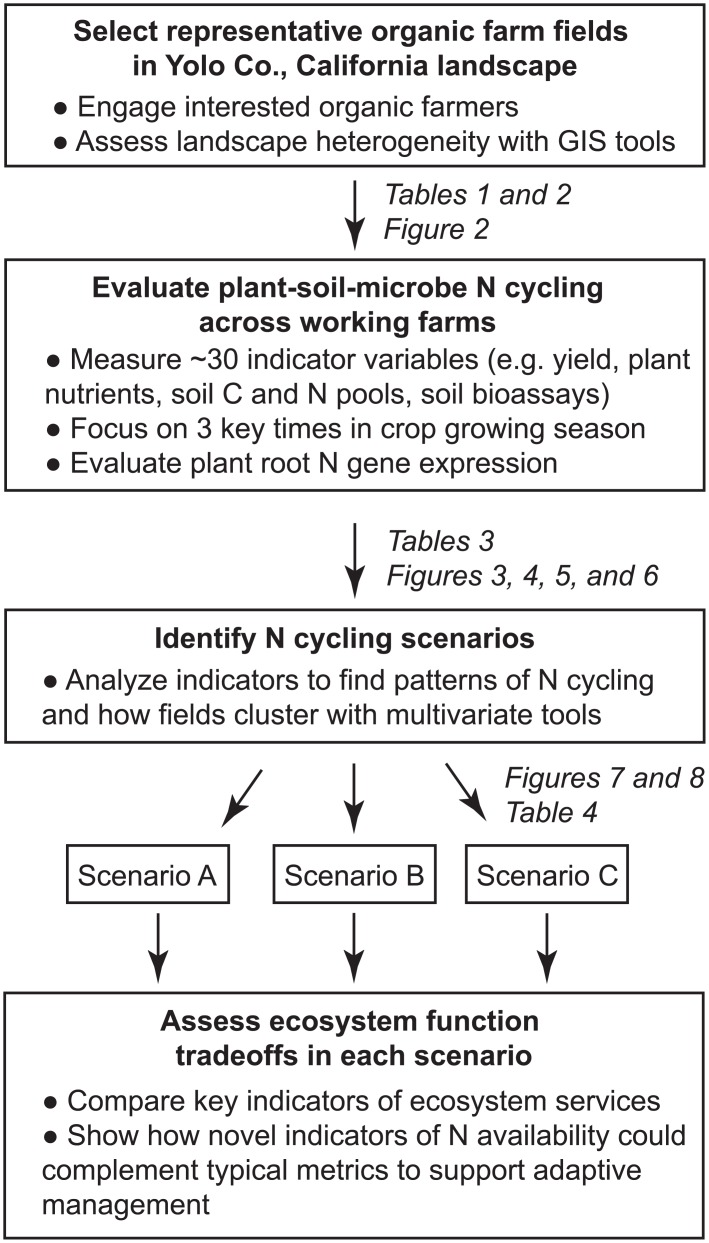
Conceptual framework of the paper. Variability across organic fields representative of a local landscape is explored to understand potential tradeoffs among ecosystem services on working farms and identify indicators of rapid N cycling to support on-farm adaptive management.

If working organic farms can achieve both tightly-coupled N cycling and high crop yields, then how do farmers do it? Are there indeed biogeochemical or plant-based indicator measures that will help organic farmers learn about their systems and provide the basis for adaptive management? Tomato (*Solanum lycopersicum* L.), a model species for plant N metabolism [35] and plant genetics [36], is widely grown on organic farms in California, where organic farmers use a variety of management practices. This provides a unique opportunity for a landscape study on how variability in SOM and management relate to yield and N cycling on working organic farms and how root expression of N metabolism genes could indicate rapid plant-soil-microbe N cycling (Fig 1). The overall hypothesis of this study is that tightly-coupled N cycling will be associated with higher levels of total and labile soil C and N and more diverse nutrient inputs (e.g. with a range of N availability). In turn, expression of root N metabolism genes will be elevated and more closely related to soil bioassays for N availability than inorganic N pools in such fields.

A landscape approach was used to assess crop yields, plant-soil N cycling, root gene expression, and the potential for soil N retention across a representative set of organic fields growing Roma-type tomatoes in one county in California, USA. High-input and high-value vegetable, fruit, and nut production dominate this landscape. The study took place during the tomato growing season to focus on the synchrony between soil N availability and crop N demand. The participatory framework in concert with GIS-based evaluation of land in organic tomato production was designed to provide real world context for evaluating novel combinations of indicators for N cycling in organic systems. The specific objectives were to: 1) identify different N cycling patterns in organic fields representative of the local landscape based on a suite of plant, soil, and soil microbial variables; 2) examine how root expression of key N metabolism genes relates to biogeochemical indicators of plant-microbe-soil N cycling; and 3) evaluate tradeoffs among ecosystem functions in N cycling scenarios.

## Methods

### Ethics statement

Permission for site access was granted by landowners. All sites were privately owned and no permits were required.

### Study region, site selection, and stakeholder engagement

**Fig 2:**
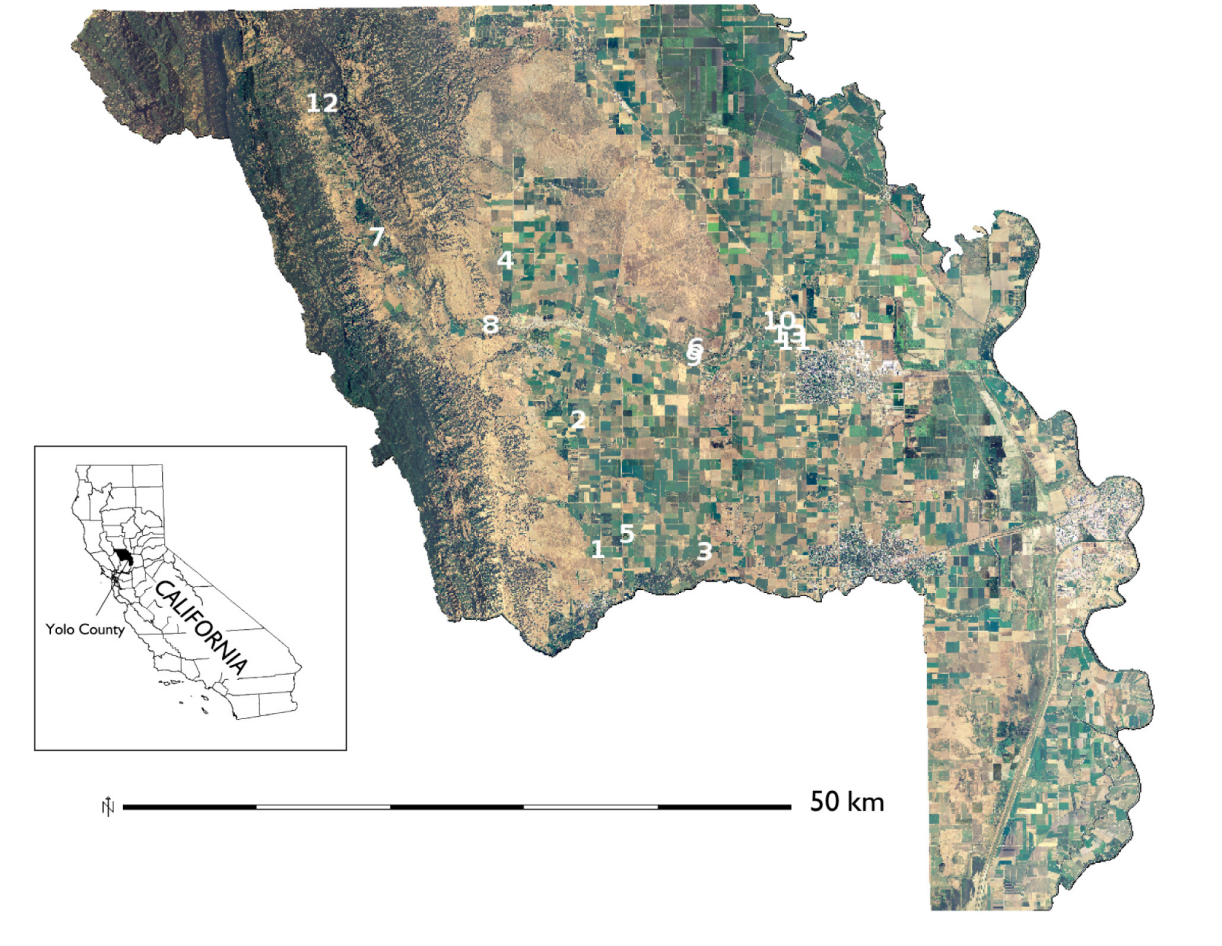
Map of the study sites. Locations of 13 organically-managed Roma-type tomato fields in Yolo Co., California, USA sampled over the 2011 growing season. Aerial imagery of the study area was derived from the National Agriculture Imagery Program (USDA-FSA-APFO NAIP).

The organically-managed fields in this study were on similar parent material (mixed alluvium) in Yolo County, California, which is situated along the western side of the Sacramento Valley (Fig 2). The Mediterranean-type climate has cool, wet winters and hot, dry summers. Annual precipitation in 2011 was 403 mm, and the mean maximum and minimum temperatures were 21.7 and 7.3°C, respectively, compared to 462 mm, 23.1°C, and 8.4°C for the previous 20 years (http://www.cimis.water.ca.gov/). Organic farming has a long history in this area (ca. 30 yrs) [37] and is relatively widespread and continuing to grow [38]. From 1989–2011, certified organic acreage in Yolo County, California increased 15-fold while production value increased nearly 30-fold to >$30M [39].

Farms growing organic Roma-type tomatoes in 2011 in Yolo County were identified using the California Certified Organic Farmers (CCOF) directory and farmers were contacted during the winter of 2010–11. CCOF is the primary organic certifier in this region of California [37]. Widespread interest among organic farmers in this region to improve N cycling and increasing concerns about N loss due to state-level policy initiatives related to greenhouse gas emissions and water quality provided an entry point to engage a variety of farmers in this study. Eight growers expressed interest in the project and identified the fields in which they expected to transplant tomatoes in early April 2011 (13 total, S1 Table). Through multiple one-on-one meetings with these farmers we learned management practices (Table 1) and following the study, we discussed biophysical and management data from their field(s) relative to data from other (anonymous) fields in the study and potential reasons for differences.

**Table 1:** Soil characteristics, soil types, and nutrient management of the 13 organic tomato fields studied in Yolo County, California, USA.

field	total C (g kg^-1^)		total N(g kg^-1^)		soil texture^a^	soil type^b^	primary organic inputs^c^	secondary nutrient inputs^d^	approx. annual N inputs^e^ (kg N ha^-1^)
	mean	se	mean	se					
1	6.7	0.17	0.8	0.03	loam	Tehama	manure	none	n.e.
2	9.6	0.23	1.2	0.02	silt loam	Tehama	manure	none	n.e.
3	10.7	0.17	1.3	0.03	silt loam	Capay	manure	none	n.e.
4	11.1	0.22	1.4	0.03	silt loam	Tehama	vetch	guano, soluble	192
5	11.2	0.22	1.4	0.03	silt loam	Capay	manure, vetch	none	202
6	12.5	0.53	1.4	0.07	silt loam	Brentwood	manure, vetch	guano	236
7	12.8	0.52	1.4	0.05	silt loam	Yolo	compost, vetch	pellets, soluble	n.e.
8	13.2	0.38	1.5	0.04	loam	Yolo	compost	pellets, soluble	221
9	13.9	0.24	1.6	0.03	silt loam	Yolo	manure, vetch	guano	236
10	16.5	0.33	1.7	0.04	silt loam	Yolo	compost	Chilean nitrate	126
11	17.1	0.48	1.8	0.04	silt loam	Yolo	compost	Chilean nitrate	126
12	18.1	0.76	2.0	0.08	loam	Yolo	compost	soluble	176
13	20.0	0.56	2.1	0.05	silt loam	Yolo	compost	Chilean nitrate	126

Table is adapted from Bowles et al. (2014).

^a^ Based on measured sand, silt, and clay content in 0–15 cm surface soil (Bowles et al. 2014).

^b^ Tehama loam: fine-silty, mixed, superactive, thermic Typic Haploxeralfs; Capay silty clay: fine, smectitic, thermic Typic Haploxererts; Brentwood silty clay loam: fine, smectitic, thermic Typic Haploxerepts; Yolo silt loam: fine-silty, mixed, superactive, nonacid, thermic Mollic Xerofluvents.

^c^ Compost and manure were applied in fall 2010, with the exception of field 5, in which manure was applied in early spring prior to tomato transplanting. Winter vetch cover crops were incorporated prior to transplanting. Compost was composted yard waste with a C:N ratio ranging from 15–18. Manure was poultry manure or poultry litter with a C:N ratio ranging from 9.8–15.

^d^ Secondary nutrient inputs are generally applied as a sidedressing or through drip line during the growing season. Guano refers to seabird guano (12-12-2.5). Pellets were pelletized poultry manure (6-3-2). Chilean nitrate (16-0-0) is NaNO_3_, a mined mineral product. Soluble refers to solubilized organic fertilizers, especially fish emulsions, which have range of nutrient concentrations.

^e^ Approximate quantities of N added through compost, manure, secondary organic fertilizers, and vetch cover crops were estimated based on farmers' reported application rates and analysis of material or based on farmers' testing records. On several fields, data were not available for every input, so these rates are considered not estimable (n.e.).

### Landscape analysis

GIS analysis of the land in organic tomato production was performed in order to ascertain how well the 13 fields that were sampled compared to the range of variability in organic tomato fields in Yolo County. Soil, landscape, and management attributes of all fields in organic tomato production in Yolo County were characterized with a landscape regionalization approach [40]. A set of 103 points were randomly assigned to all such fields based on a 2008 field-scale county survey [41], representing one point every 4 hectares. For each of these points, the values of 12 variables were compiled from several sources. Categorical variables included soil great group and soil drainage class from the SSURGO database [42], the number of crop rotation types in a one mile surrounding square, and an agricultural sub-region classification [41]. Continuous variables from the SSURGO database included percent sand, silt, and clay, organic matter, elevation, and the Storie index (a measure of agricultural productivity). Distance to natural vegetation was obtained from a land cover map of California [43].

GIS data were subjected to a clustering algorithm, partitioning around medoids (PAM), based on a distance matrix derived from Gower’s dissimilarity algorithm [40,44]. PAM analysis with five clusters returned the best defined clusters yielding an average silhouette width (si) of 0.499. The proportion of the landscape in organic tomato production represented by each cluster was calculated by performing a Voronoi tessellation of the 103 points, assigning each polygon of the tessellation to a cluster type, and then intersecting the tessellation with the field boundaries to allow determination of cluster areas. Based on a lack of grower interest, cluster 2 was not represented.

### Soil and plant field sampling

Soil and plant sampling was designed to capture indicators of ecosystem functions related to plant-soil N cycling at times corresponding to key agronomic and phenological events, including immediately prior to tomato transplanting (pre-transplant), peak tomato growth period (mid-season), and tomato harvest (harvest). In each field, plots (90 m^2^) were established at six random locations within a 0.25 ha area.

Pre-transplant measurements took place several days prior to tomato transplanting but after other field operations, such as tillage, incorporation of organic amendments and/or vetch cover crops, and bed formation. Tomatoes were transplanted in all fields between 6 April and 20 April, 2011. In each of the six plots, three soil cores (6.3 cm in diameter) for each depth (0–15, 15–30, and 30–75 cm) were removed from tomato beds and composited in the field, separately for each plot.

For mid-season measurements, fields were all sampled within two weeks of one another, an average of 68 days after transplanting. This corresponds to the mid- to late-bloom stage when tomato N demand is maximal [45]. A soil core (15 cm in diameter and 0–15 cm deep) was removed in each plot, situated between two tomato plants 15 cm from the planting row. Three 50–150 mg (fresh weight) subsamples of roots were promptly removed from the soil core in the field under minimized/indirect light, rinsed, patted dry, and flash frozen in liquid nitrogen for subsequent RNA extraction (see below). Deeper soil cores (15–30 and 30–75 cm deep) were removed from the same hole. The two plants adjacent to this core were cut at the base and petiole samples from recently matured leaves were removed. Plants were rinsed and dried at 60°C for two weeks before grinding and analyzing for C and N (see below).

Tomato yields were sampled just before the farmer’s harvest. In each plot (six per field), two 1m × 2m sub-plots were established. At each of these subplots, individual tomato plants were cut at the base and ripe fruit (i.e. harvestable) was separated by hand from green and decayed fruit (i.e. unharvestable). This process uses criteria similar to that of machine harvested tomatoes as well as those harvested by hand for fresh market sales. Biomass of fruits and shoots were weighed in the field (fresh weight) then subsampled and dried at 60°C for 2 weeks, before grinding and analyzing for C and N (see Section 2.4). Soil cores (0–15, 15–30, and 30–75 cm depths) were also taken from each subplot and composited in the field for measurements described below.

### Laboratory processing of soil and plant samples

Soil samples were kept on ice and processed within several hours of field extraction by thoroughly homogenizing by hand. Soils from the 0–15 cm depth were analyzed for a variety of soil C and N fractions, bioassays for N availability, and soil properties, while deeper depths (15–30 and 30–75 cm) were analyzed for inorganic N and gravimetric water content (GWC) only. Inorganic N was extracted from moist soils with 2M KCl and analyzed colorimetrically for NH_4_^+^ and NO_3_^-^ [46,47]. Potentially-mineralizable N (PMN) was measured as NH_4_^+^ liberated during a seven-day anaerobic incubation at 37°C [48]. Chloroform fumigation-extraction followed by UV-persulfate oxidation and alkaline persulfate oxidation was used to measure microbial biomass C (MBC) and N (MBN), respectively [49,50]. K_2_SO_4_ extractable organic C (EOC) and N (EON) were quantified in non-fumigated samples [51]. Permanganate oxidizable C (POXC), which reflects a processed soil fraction that is sensitive to management was measured according to standard procedures [25]. Gravimetric water content was determined by drying at 105°C for 48 h. Air dried soil samples were sieved to 2 mm, ground, and analyzed for total C and N at the UC Davis Stable Isotope Facility. Methods and results for other soil measurements (e.g. texture and pH) are presented in previous work [10].

Shoots and fruit were analyzed for total C and N, δ^13^C, and δ^15^N at the UC Davis Stable Isotope Facility. Petiole NO_3_^-^, an indicator of recent N status in conventionally-produced vegetables [52], was measured in the most recently-matured leaves [53]. Petiole NO_3_^-^ changes rapidly with growth stage, so the data are graphed by post-transplanting growing degree day [54] to account for phenological differences among fields as a result of slightly different sampling times relative to transplanting.

### Root RNA purification and quantitative real-time RT-PCR

Root RNA was extracted using Trizol reagent (Invitrogen, Carlsbad, CA) according to the manufacturer’s guidelines followed by DNase digestion using RQ1 RNase-free DNase (Promega, Madison, WI). Total RNA was purified using the RNeasy Plant Mini Kit (Qiagen Sciences, Germantown, MD). RNA concentrations and quality were assessed using the Agilent Nanodrop and the RNA 6000 Nano Assay (Bioanalyzer 2100, Agilent, Santa Clara, CA). Only RNA samples with RNA integrity numbers (RIN) of at least 7.0 were used for subsequent analyses. These RNA were used for cDNA synthesis for qRT-PCR analysis. cDNA was synthesized from 0.5 μg DNase-treated total RNA using the Superscript III kit (Invitrogen, Carlsbad, CA).

Quantitative real-time RT-PCR (qRT-PCR) was performed as described in previous work [30,31], using the primer pairs tested and reported therein and using a StepOnePlus Real-Time PCR system (Applied Biosystems, Foster City, CA). Seven key genes involved in root N uptake and assimilation that had previously been shown to be responsive to an N pulse in an organic soil were assessed [30,31]: high-affinity NH_4_^+^ transporters AMT1.1 and AMT1.2 (XM_004247726; NM_001247324); high-affinity NO_3_^-^ transporter NRT2.1 (AF092655); nitrite reductase Nii (XM_004230772); cytosolic and plastidic glutamine synthetases GS1 and GS2 (XM_004236638; XM_010324491); and NADH-dependent glutamate synthase NADH-GOGAT (XM_004234907). The tomato actin (BT013707) and ubiquitin genes (X58253) were used as reference control genes as they did not exhibit differential expression among N treatments in previous field experiments [30,31]. Relative expression was analyzed according to the ΔΔCT method with multiple reference control genes [55] and using inter-run calibration [56]. Each gene was mean-centered and log transformed to help correct positive skewing.

### Statistical analyses

Means for variables in all tables, figures, and the text are expressed with 95% confidence intervals (*x* ± 95% CI; n = 6 for field-level summaries). CIs can assist with means comparisons based on the “inference by eye” method [57]. Roughly, 95% CIs that overlap with another mean are not different but when the overlap of intervals is no more than half of one interval arm, then the means are different at *p* ≈ 0.05. Intervals that barely touch are significant at *p* ≈ 0.01 and intervals that are separated by at least half of one interval arm are different at *p* ≈ 0.001. Soil NH_4_^+^ and NO_3_^-^ showed positive skewing and thus were log transformed prior to calculation of means and CIs. Back-transformed means and 95% CIs are shown for these variables. Fields were clustered based on 28 plant, soil, and microbial variables using the *k*-means method implemented in R. The optimal number of groups (three) chosen was based on the Calinski-Harabasz criterion and by examining sums of squared error scree plots [58]. *F*-statistics were calculated for each variable based on their cluster grouping to assess the relative magnitude of the “cluster effect”, i.e. higher *F*-statistics indicate more differentiation among clusters for a given variable [10].

## Results

### Field and landscape characteristics

The 13 organically-managed Roma-type tomato fields spanned a three-fold range of soil C (6.7–20.0 g kg^-1^) and N (0.8–2.1 g kg^-1^) and had similar soil texture, soil types, and soil pH (Table 1). Field numbers are in order of increasing total soil C. Variation in nutrient inputs, including highly-labile secondary inputs (e.g. seabird guano and Chilean nitrate) indicated diverse and intensive organic management strategies across these farms (Table 1).

The 13 fields encompassed the majority of the variation (≈65%) in the focal landscape (the total area in organic tomato production in Yolo County, California), i.e. all but one of the five clusters, or landscape types, identified by GIS and multivariate analysis were represented (Table 2). Thus, a range of soil characteristics representative of this region was accounted for by the fields sampled. Other characteristics, such as the low number of crop rotation types (based on a 2008 field-scale county survey [41]) differed little across the clusters and reflect the intensity of agricultural management in this region.

**Table 2:** Medoid values of the 12 GIS variables for each of five clusters, i.e. landscape types.

Variable	Cluster 1	Cluster 2	Cluster 3	Cluster 4	Cluster 5
*medoid values of clusters*					
Sand content (%)	10.5	7.9	5.5	11.4	30.2
Silt content (%)	66.3	64.8	47.0	51.1	43.3
Clay content (%)	23.2	27.2	47.5	37.6	26.5
Organic matter content (%)	1.58	1.20	1.41	0.38	0.38
Soil great group	Xerorthents	Haplaquepts	Chromoxererts	Xerochrepts	Haploxeralfs
Elevation (m)	67.8	4.4	55.0	37.6	79.6
Drainage class	5	3	4	5	5
Storie index^a^	100	27	50	81	72
Crop rotation^b^	1	1	1	1	2
Distance to natural vegetation (km)	8	15	5	8	23
Crop class^c^	unspecified vegetables	processing tomatoes	processing tomatoes	processing tomatoes	unspecified tomatoes
Yolo Region^d^	Capay Valley	Yolo Bypass	Yolo West	Hungry Hollow	Hungry Hollow
*spatial characteristics of each cluster*					
Hectares^e^	251.7	548.2	152.6	288.1	304.8
Proportion of landscape^f^ (%)	16.3	35.5	9.9	18.6	19.7
Fields sampled	7, 9, 10, 11, 12, and 13	none available to sample	1, 3, and 5	6	2, 4, and 8

Variables were derived from publicly available databases (e.g. SSURGO). Also shown are spatial characteristics of each cluster and sampled fields that were part of each cluster. Values were derived from Partitioning Around Medoids analysis on the 103 random points assigned to land in organic tomato production in Yolo County, California, USA, i.e. the landscape of study. Soil variables are from the 0–100 cm depth.

^a^ A method of soil rating that summarizes the land's potential utilization and productive capacity. Grade 1 (excellent): 100–80; Grade 2 (good): 79–60; Grade 3 (fair): 59–40; Grade 4 (poor): 39–20.

^b^ Number of crop rotation types in a 1-mile surrounding square, based on 2008 survey by Richter et al. (2009)

^c^ Typical crop grown, based on 2008 survey by Richter et al. (2009)

^d^ Agricultural region of Yolo County, as defined by Richter et al. (2009)

^e^ Number of ha represented by the cluster, based on the tesselation analysis of the land in organic tomato production.

^f^ Proportion of the total landscape area (i.e. land in organic tomato production in Yolo Co., California) represented by the cluster

### Soil inorganic N pools and microbial biomass

**Fig 3:**
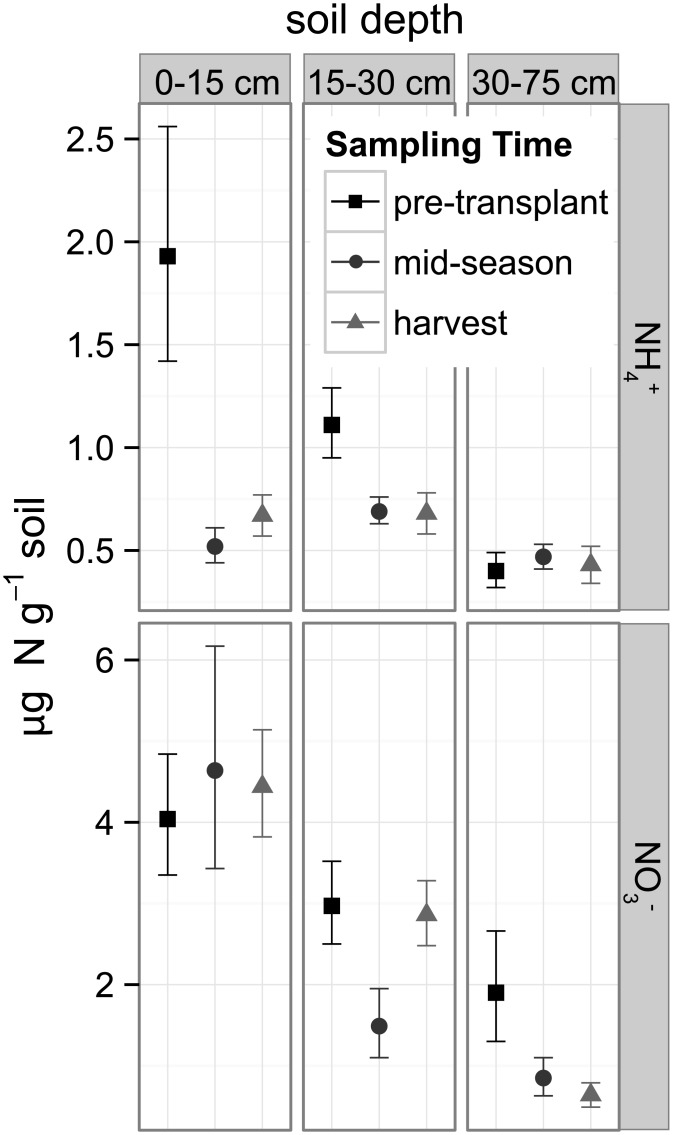
Soil ammonium and nitrate (NH_4_^+^ and NO_3_^-^) at three depths and three sampling times. Data are combined from 13 organically-managed Roma-type tomato fields in Yolo Co., California, USA. The three sampling times corresponded to key agronomic and phenological events. Shown are back-transformed means and 95% confidence intervals.

**Fig 4:**
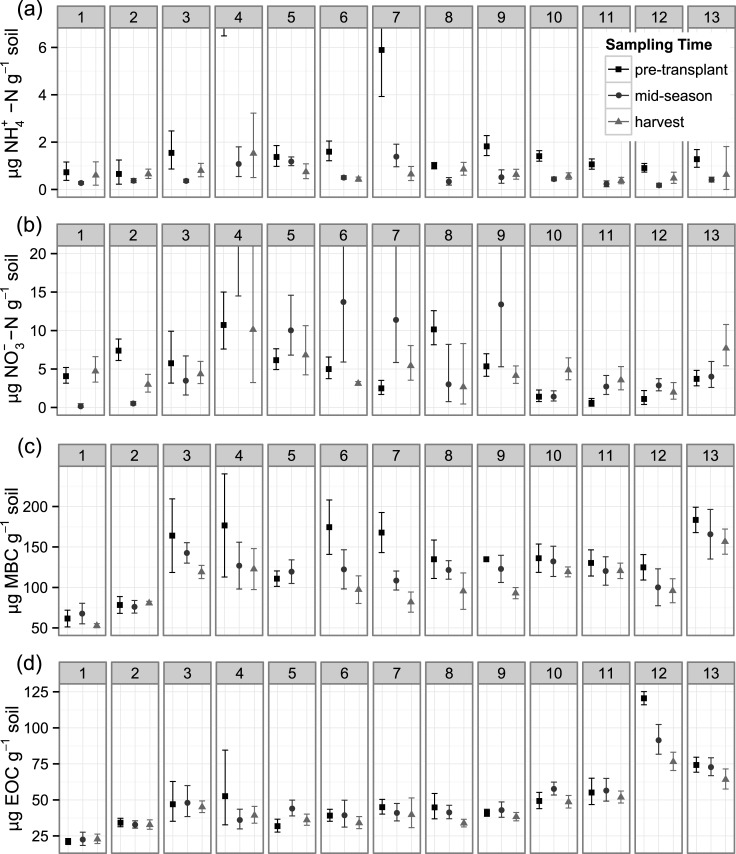
Soil inorganic N, microbial biomass C (MBC) and K_2_SO_4_ extractable organic C (EOC) from surface soil (0–15 cm depth). Soils were sampled at three times across 13 organically-managed Roma-type tomato fields in Yolo Co., California, USA. For ammonium and nitrate (NH_4_^+^-N and NO_3_^-^-N), shown are back-transformed means and 95% confidence intervals. For MBC and EOC, shown are means and 95% confidence intervals. In order to increase resolution of the majority of the data, two means are not shown, both from field 4. NH_4_^+^-N at pre-transplant in field 4 was 30.5 μg N g^-1^ (6.5 < μ < 131.8) and NO_3_^-^-N at mid-season in field 4 was 35.0 μg N g^-1^ (14.5 < μ < 82.7).

Soil NH_4_^+^ and NO_3_^-^ pools were highly variable across fields, sampling times, and depths (Figs 3 and 4; S2 Table). For instance, soil NO_3_^-^ at mid-season (when tomato N demand is highest) ranged from a low of 0.2 μg N g^-1^ (0.0 < μ < 0.5; mean ± 95% CI) in field 1 to a high of 35.0 μg N g^-1^ (14.5 < μ < 82.7) in field 4 (Fig 3), which also had very high pre-transplant levels of NH_4_^+^. In general, surface soil NO_3_^-^ concentrations tended to be lower and less variable at the low and high ends of the soil C gradient (e.g. fields 1, 2, 10, 11, 12, and 13) relative to fields in the middle of the gradient (Fig 4). Considering all fields collectively, the mean surface soil NO_3_^-^ concentration was only 4.6 μg N g^-1^ (3.4 < μ < 6.2) at midseason, which was similar to the values at transplant and harvest (Fig 3). Soil NO_3_^-^ declined significantly with depth, with the lowest mean value of 0.6 μg N g^-1^ (0.5 < μ < 0.8) at harvest in the 30–75 cm depth. For the individual fields, soil NH_4_^+^ was less variable than NO_3_^-^ in the 0–15 cm depth (Fig 4). Considering all fields collectively, soil NH_4_^+^ declined significantly from pre-transplant to mid-season sampling at both the 0–15 cm and 15–30 cm depths and then remained similar at harvest (Fig 3).

Most fields had similar MBC for a given sampling time (Fig 4), with the exception of fields 1 and 2, which were substantially lower than the overall mean of 119.8 ± 5.0 μg C g^-1^ (mean ± 95% CI). These fields also had the lowest total soil C (field numbering is in order of increasing total soil C; see methods). Collectively across fields, mean MBC declined over the course of the season, from a high of 137.6 ± 10.2 μg C g^-1^ at the pre-transplant sampling to 117.4 ± 6.7 μg C g^-1^ and 102.9 ± 6.7 μg C g^-1^ at the mid-season and harvest sampling, respectively; however, these declines varied by field, with some fields (e.g. 3, 6, 7, 8, and 9) showing stronger declines than others. Patterns of EOC were similar to those of MBC (Fig 4), with the exception of field 12, which had high values relative to other fields, particularly at the pre-transplant sampling.

### Tomato nitrogen, isotopic ratios, and yield

**Fig 5:**
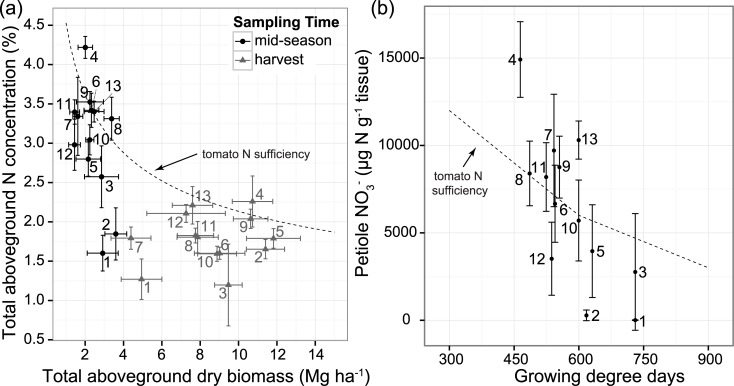
Two measures of tomato N status. Plants were sampled from 13 organically-managed Roma-type tomato fields in Yolo Co., California, USA. a) Whole plant N concentration at two sampling times (mid-season and harvest) relative to the critical N concentration for processing tomatoes (dotted line, N_c_ = 45.3×dry biomass^-0.327^ [68]); and b) petiole NO_3_^-^ concentration at mid-season sampling. Since petiole NO_3_^-^ rapidly changes, concentrations are given in terms of growing degree days to correct for slight variations in sampling time in each field. The dotted line shows current tissue sufficiency guidelines for petiole NO_3_^-^ based on conventional processing tomato production [54].

Measures of tomato N sufficiency varied widely across the 13 organic fields, ranging from deficient to luxury [45] N levels. Total aboveground N concentration at midseason overlapped or fell slightly below the critical N concentration for processing tomatoes in most fields (i.e. the minimum N concentration required for maximum plant growth at a given growth stage), with N concentrations between 2.5 and 3.5% (Fig 5a). Exceptions were fields 1 and 2 which were markedly lower (1.60 ± 0.23% and 1.85 ± 0.33%, respectively) and field 4, which was higher (4.22 ± 0.14%). The same general pattern occurred for the harvest sampling. Petiole NO_3_^-^ concentration in four fields overlapped the sufficient concentration, based on published guidelines (Lorenz and Tyler, 1983), while five fell below it, and four rose above it (Fig 5b). Petiole NO_3_^-^ was especially high in field 4. Petiole NO_3_^-^-N showed a broadly similar pattern to total aboveground N concentration, as reflected in the strong linear relationship between them (*p* < 0.0001, R^2^ = 0.81).

At the mid-season sampling, shoot δ^15^N ranged from 4.22 ± 0.65‰ in field 10 to 13.29 ± 1.18‰ in field 6. Fields 3, 4, 6, and 9 had the highest shoot δ^15^N, all above 12‰, and all but field 3 used seabird guano. Fields 8, 10, 11, and 13 had the lowest shoot δ^15^N, close to 4‰, and all but field 8 used Chilean nitrate.

**Fig 6:**
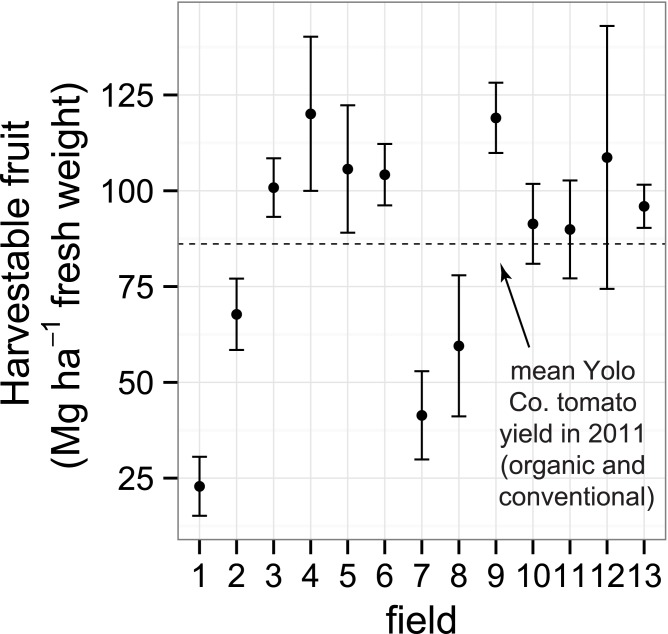
Harvestable tomato fruit yield (fresh weight). Yields were measured from 13 organically-managed Roma-type tomato fields in Yolo Co., California, USA. Shown are means and 95% confidence intervals. The dotted line represents the overall Yolo County average processing tomato yield in 2011 (86.1 Mg ha^-1^), including both conventional and organic tomato production.

Mean harvestable fruit yield across all 13 fields was 86.7 ± 7.2 Mg ha^-1^ (fresh weight) and was similar to the overall Yolo County mean 2011 tomato yield (86.1 Mg ha^-1^), which included both conventional and organic fields (Fig 6). Field 4 had the highest yield overall (120.1 ± 20.1 Mg ha^-1^) followed closely by field 9 (119.0 ± 9.2 Mg ha^-1^), and field 1 had the lowest (22.9 ± 7.7 Mg ha^-1^). Nine of 13 fields had means higher than the county average, and six of these fields were significantly higher. There was also substantial variability in tomato aboveground biomass and N content at harvest across fields (S3 Table), which largely reflected the pattern of fresh weight yields. For instance, total aboveground N ranged from 64 kg N ha^-1^ in field 1 to 243 kg N ha^-1^ in field 4 with a mean across all fields of 154 kg ha^-1^.

### Expression of N metabolism genes in plant roots

Expression of cytosolic glutamine synthetase GS1 in roots was more strongly related to indicators of plant-soil N cycling than were the other six key genes involved in root N metabolism (Table 3). Of the soil variables, GS1 was more strongly related to soil bioassays for N availability than to inorganic N pools (Table 3). Microbial biomass N and PMN were most strongly associated with expression of GS1 in roots, followed by soil NO_3_^-^. Permanganate oxidizable C and MBC, both indicators of labile soil C pools, also had significant associations with GS1 expression in roots, but soil NH_4_^+^ did not. Expression of GS1 also was positively associated with shoot N and petiole NO_3_^-^, as was glutamate synthase NADH-GOGAT. Inclusion of GWC as a covariate in multiple linear regression models improved the proportion of explained variation in GS1 expression (e.g. an increase in adjusted R^2^ from 0.22 to 0.30 for MBN and GWC together vs. microbial biomass N alone) (S1 Fig).

**Table 3:** Pearson correlation coefficients between root expression of key N metabolism genes^a^ and plant and soil variables^b^ measured across 13 organic Roma-type tomato fields in Yolo Co., California, USA.

N metabolism genes^a^	Shoot N percent	petiole NO_3_^-^	soil NH_4_^+^	soil NO_3_^-^	PMN	MBC	MBN	EOC	EON	POXC	GWC
AMT1.1	**-0.28**	-0.15	-0.07	-0.22	-0.06	-0.02	-0.15	-0.04	0.07	**-0.29**	0.13
AMT1.2	0.22	0.20	0.20	**0.27**	-0.03	0.03	**0.27**	-0.15	-0.16	-0.08	0.20
NRT2.1	**-0.26**	**-0.32**	-0.08	-0.13	-0.05	-0.06	-0.19	0.14	**0.24**	-0.13	-0.03
Nii	**-0.27**	**-0.34**	-0.01	-0.09	-0.19	-0.09	-0.15	-0.11	0.02	-0.24	-0.13
GS1	**0.47**	**0.44**	0.19	**0.34**	**0.37**	**0.31**	**0.45**	0.11	0.09	**0.24**	**0.43**
GS2	-0.17	-0.20	0.09	-0.02	-0.13	**-0.36**	-0.21	-0.06	0.02	-0.17	-0.17
NADH-GOGAT	**0.39**	**0.34**	**0.27**	**0.41**	0.08	0.22	0.09	0.14	0.17	0.10	0.20

All soil variables are from 0–15 cm surface soil. For units of each variable, see Table 4. Correlations significant at *p* < 0.05 or less are in bold.

^a^ AMT1.1 and AMT1.2: high-affinitity NH_4_^+^ transporters; NRT2.1: high-affinity NO_3_^-^ transporter; Nii: nitrite reductase; GS1: cytosolic glutamine synthetase; GS2: plastidic/chloroplastic glutamine synthetase; NADH-GOGAT: glutamate synthase.

^b^ PMN: potentially mineralizable N; MBC: microbial biomass C; MBN: microbial biomass N; EOC: extractable organic C; EON: extractable organic N; POXC: permanganate oxidizable C; GWC: gravimetric water content.

### Multivariate analysis of yield, N cycling, and root gene expression

**Fig 7:**
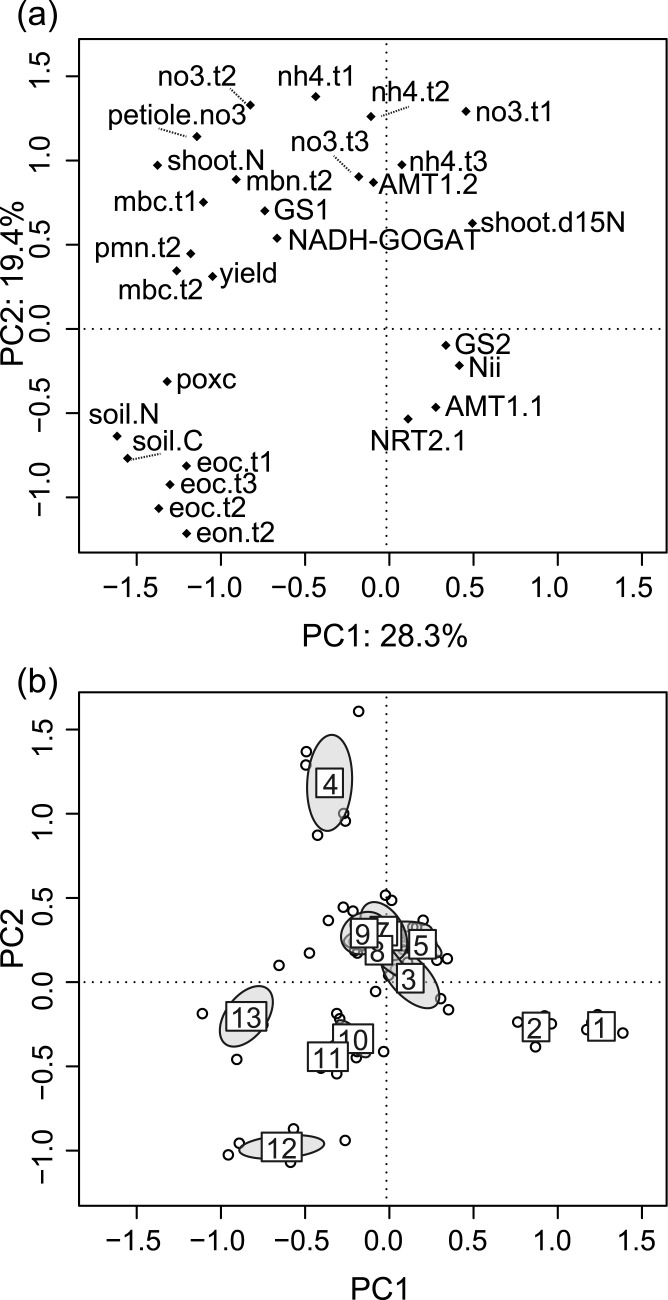
Principal components analysis of soil C and N pools, soil bioassays, tomato yield and N, and expression of key N metabolism genes in roots. Samples are from surface soil (0–15 cm) and adjacent plants from 13 organic Roma-type tomato fields in Yolo Co., California, USA. Axes 1 and 2 account for 28.3 and 19.4% of the total variation, respectively. (a) Variable loading plot. Appended suffixes “t1”, “t2”, and “t3” refer to sampling time: pre-transplant, mid-season, and harvest, respectively. (b) Sample scores with 95% confidence ellipses for fields, numbered 1–13 (6 samples per field). N metabolism genes include: high-affinitity NH_4_^+^ transporters AMT1.1 and AMT1.2; high-affinity NO_3_^-^ transporter NRT2.1; nitrite reductase Nii; cytosolic glutamine synthetase GS1; plastidic/chloroplastic glutamine synthetase GS2; glutamate synthase NADH-GOGAT.

PCA of 28 indicators of yield and plant nutrient status, root N metabolism, and soil C and N cycling (see list in Table 4) showed strong relationships among suites of variables, which clearly differentiated fields along the first two principal components (Fig 7). The first principal component explained 28.3% of the variation; on the left side of the biplot are higher values of most variables, including yield, soil bioassays, expression of root GS1 and NADH-GOGAT, and labile and total soil C and N pools (Fig 7a). Soil NH_4_^+^ and NO_3_^-^ concentrations from all three sampling times as well as AMT1.2 were associated with one another and with positive values along principal component 2, which explained 19.4% of the variation. Total soil C and N were strongly associated with EOC and EON, the soil C:N ratio, and POXC. These variables had negative values along axis 2 and thus contrasted with the pattern of soil inorganic N. Weak loading of AMT1.1, NRT2.1, Nii, and GS2 on the first two principal components reflects the lack of association of expression levels of these genes with biogeochemical and plant variables.

**Table 4:** Means and 95% confidence intervals of variables in each group of fields identified with *k*-means cluster analysis based on values of 28 plant, soil^a^, and root gene expression^b^ variables measured across 13 organic Roma-type tomato fields in Yolo Co., California, USA.

		*Group*	*1*		*2*		*3*		
		Fields included	1, 2		3, 4, 5, 6, 7, 8, 9		10, 11, 12, 13		
variable	time	units	mean	95% CI	mean	95% CI	mean	95% CI	*F* statistic
yield	harvest	Mg ha^-1^	45.3	15.7	93.0	9.7	96.5	7.9	17.0
shoot N	anthesis	%	1.7	0.2	3.3	0.2	3.2	0.1	59.2
shoot δ^15^N	anthesis	δ^15^N	10.6	1.3	9.3	1.1	5.5	0.9	16.1
petiole NO_3_^-^	anthesis	μg N g^-1^	142	154	7880	1363	6930	1317	21.1
soil NH_4_^+^	transplant	μg N g^-1^	0.7	0.3	9.3	6.8	1.2	0.1	8.5
soil NH_4_^+^	anthesis	μg N g^-1^	0.3	0.1	0.8	0.2	0.3	0.1	16.1
soil NH_4_^+^	harvest	μg N g^-1^	0.7	0.3	0.9	0.2	0.6	0.3	3.2
soil NO_3_^-^	transplant	μg N g^-1^	5.8	1.3	6.7	1.1	1.8	0.6	45.5
soil NO_3_^-^	anthesis	μg N g^-1^	0.4	0.2	16.2	6.1	2.9	0.6	45.6
soil NO_3_^-^	harvest	μg N g^-1^	4.0	1.1	6.2	1.9	4.7	1.2	0.9
PMN	anthesis	μg N g^-1^	3.1	1.3	13.3	1.7	13.7	1.8	24.6
MBC	transplant	μg C g^-1^	69.9	8.2	151.9	12.1	143.7	11.8	29.7
MBC	anthesis	μg C g^-1^	71.9	6.7	123.5	5.8	129.6	13.4	29.0
MBN	anthesis	μg C g^-1^	5.9	1.4	15.6	1.8	13.5	2.3	16.0
EOC	transplant	μg C g^-1^	27.7	4.6	44.2	4.3	75.0	12.2	30.1
EOC	anthesis	μg C g^-1^	27.9	4.0	42.3	2.3	69.9	6.8	76.8
EOC	harvest	μg C g^-1^	27.9	3.8	38.3	1.9	60.4	5.2	75.7
EON	anthesis	μg N g^-1^	3.9	0.8	6.5	0.8	13.5	1.3	82.1
POXC	anthesis	μg C g^-1^	411	35	563	30	619	39	21.6
soil C	anthesis	g kg^-1^	8.2	1.0	12.2	0.4	17.9	0.8	176.6
soil N	anthesis	g kg^-1^	1.0	0.1	1.4	0.0	1.9	0.1	130.1
AMT1.1	anthesis	ln(relative expression)	0.90	0.23	0.67	0.09	0.76	0.11	3.5
AMT1.2	anthesis	ln(relative expression)	0.57	0.22	0.84	0.10	0.58	0.11	7.6
NRT2.1	anthesis	ln(relative expression)	0.88	0.20	0.74	0.12	0.81	0.19	0.6
Nii	anthesis	ln(relative expression)	1.06	0.29	0.88	0.22	0.80	0.36	0.5
GS1	anthesis	ln(relative expression)	0.35	0.13	0.90	0.14	0.78	0.16	8.9
GS2	anthesis	ln(relative expression)	0.88	0.20	0.69	0.12	0.67	0.12	1.6
NADH-GOGAT	anthesis	ln(relative expression)	0.58	0.12	0.74	0.06	0.74	0.06	4.0

^a^ PMN: potentially mineralizable N; MBC: microbial biomass C; MBN: microbial biomass N; EOC: extractable organic C; EON: extractable organic N; POXC: permanganate oxidizable C; GWC: gravimetric water content.

^b^ AMT1.1 and AMT1.2: high-affinitity NH_4_^+^ transporters; NRT2.1: high-affinity NO_3_^-^ transporter; Nii: nitrite reductase; GS1: cytosolic glutamine synthetase; GS2: plastidic/chloroplastic glutamine synthetase; NADH-GOGAT: glutamate synthase.

Non-overlapping confidence ellipses for seven out of 13 fields on the PCA biplot indicated distinct N cycling patterns (Fig 7b). Fields 1 and 2, with the highest values along axis 1, had low values of all variables included in the analysis. Field 4 had the highest values along axis 2 corresponding with higher soil NH_4_^+^ and NO_3_^-^. Fields 10, 11, 12, and 13 were associated with high values of labile and total soil C and N. Overlapping confidence ellipses of fields 3, 5, 6, 7, 8, and 9 close to the origin indicate similar, moderate values of this suite of variables for these fields.

**Fig 8:**
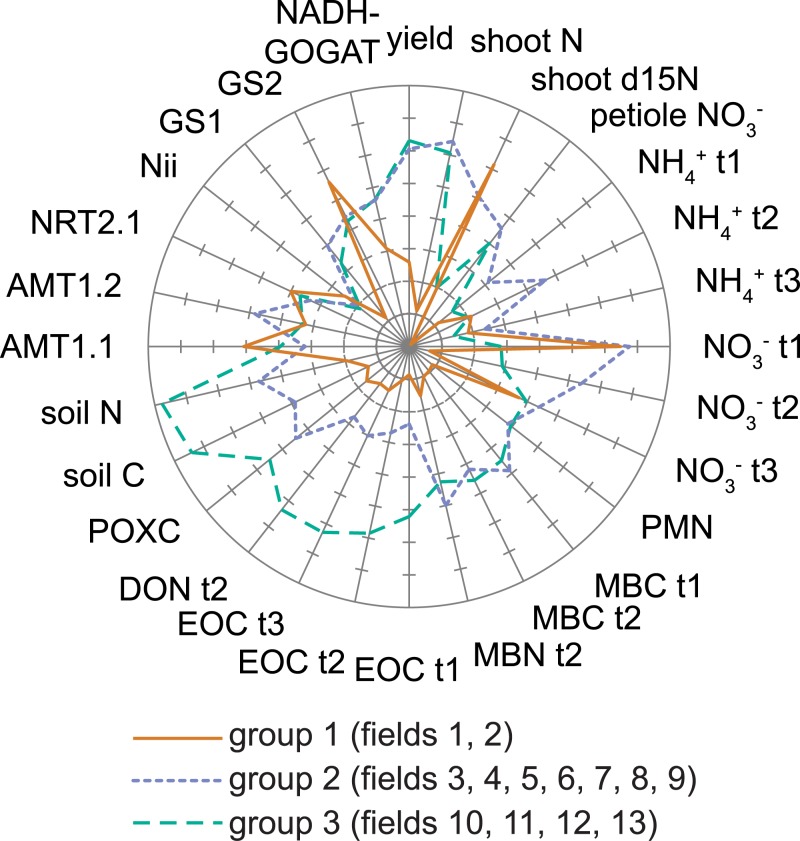
N cycling scenarios across organic fields in an intensively-managed agricultural landscape. Groups are based on *k*-means analysis of the 28 variables shown, including soil C and N pools, soil bioassays, tomato yield and N, and expression of key N metabolism genes in roots, across 13 fields in Yolo Co., California, USA. Each variable was normalized on a 0–1 scale, and then a mean for each cluster was calculated for each variable. N metabolism genes include: high-affinitity NH_4_^+^ transporters AMT1.1 and AMT1.2; high-affinity NO_3_^-^ transporter NRT2.1; nitrite reductase Nii; cytosolic glutamine synthetase GS1; plastidic/chloroplastic glutamine synthetase GS2; glutamate synthase NADH-GOGAT.

Three groups of fields were identified by *k*-means cluster analysis of the same 28 variables included in the PCA (Table 4 and Fig 8). Group 1 included fields 1 and 2, which had low mean values for yield (45.7 Mg ha^-1^), the lowest mean soil C and N (8.2 and 1.0 g kg^-1^, respectively) and soil inorganic N pools (e.g. 0.4 μg NO_3_^-^-N g^-1^ at anthesis), and the lowest mean value of GS1 relative expression in roots. Groups 2 and 3 had similarly higher mean yield (93 and 96.5 Mg ha^-1^, respectively), shoot N, and petiole NO_3_^-^ than group 1, but these two groups differed substantially in their soil C and N pools. Group 2 had higher soil NH_4_^+^ and NO_3_^-^ pools as well as root expression of AMT1.2 while group 3 had higher total and labile soil C pools. Expression of GS1 was similar in both groups. Based on the relative magnitude of *F*-statistics calculated for each variable, soil C and N, EOC, EON, shoot N, and soil NO_3_^-^ at transplant and anthesis were most strongly differentiated across the three groups. The high *F*-statistics of AMT1.2 and GS1 relative to other N metabolism genes indicate that root expression of these genes are most responsive to soil N cycling.

## Discussion

This study confirms that working organic farms can produce high yields with tightly-coupled N cycling that minimizes the potential for N losses. Such farms had the highest soil C and N and used high C:N organic matter inputs coupled with labile N inputs that resulted in high soil biological activity, low soil inorganic N pools, high expression for a root N assimilation gene, adequate plant N, and high yields. Organic systems trials have previously shown crop N deficiencies [59–62] that lead to less-than-ideal crop productivity [63]; losses of N when N availability is poorly synchronized with crop N demand [64,65]; or alternatively, that organic production can reduce N losses [4]. But how working organic farms achieve yields competitive with high-input conventional production (in a top tomato producing county in California [66]) with low potential for N losses has not been demonstrated. Elevated expression of a key gene involved in root N assimilation, cytosolic glutamine synthetase GS1, in fields with tightly-coupled N cycling confirmed that plant N assimilation was high when plant-soil-microbe N cycling was rapid and inorganic N pools were low, thus showing potential as a novel indicator of N availability to plants. Improving biologically-based farming systems will benefit from research that uses novel tools to uncover innovations happening on farms, especially if the research process helps facilitate knowledge exchange among farmers and researchers.

### Three “scenarios” of N cycling

To characterize the substantial variation in crop yield, plant-soil N cycling, and root gene expression across 13 fields growing the same crop on similar soil types, we propose three N cycling scenarios: “tightly-coupled N cycling”, “N surplus”, and “N deficient”. Values of indicator variables suggest differing levels of provisioning, regulating, and supporting ecosystem services in each scenario (Table 5).

**Table 5:** Ecosystem functions representing ecosystem services, indicators of processes or stocks, and the indicator variables measured across 13 organic Roma-type tomato fields in Yolo Co., California, USA.

Ecosystem service	Ecosystem service type	ecosystem functions (processes or stocks)	Indicator variables for each ecosystem function
food production	provisioning	crop yield	tomato fruit biomass
soil, water, and air quality	regulating	N retention	soil NO_3_^-^ concentration (-)^a^
		soil organic matter quantity and quality	total soil C and N; permanganate oxidizable C (POXC); extractable organic C and N
nutrient cycling	supporting	N mineralization	potentially mineralizable N (PMN)
		plant N uptake	shoot N; petiole NO_3_^-^; expression of N uptake and assimilation genes
		microbial activity	microbial biomass C and N

^a^ Lower values of the variable is indicative of higher indicator processes

Fields in group 3 show evidence of tightly-coupled plant-soil N cycling, a desirable scenario in which crop productivity is supported by adequate N availability but low potential for N loss. Despite consistently low soil NO_3_^-^ pools in these fields, well below the critical (i.e. responsive to N fertilizer) mid-season level for conventional processing tomatoes in California (~16 μg NO_3_^-^-N g^-1^ soil) [67], total aboveground N concentrations were very close to or only slightly below the critical N concentration for processing tomatoes [68]. Tomato yields were also above the county average (96.5 Mg ha^-1^ for group 3 vs. 86.7 Mg ha^-1^ for Yolo Co.).

This discrepancy between low soil inorganic N pool sizes and adequate tomato N status is due to N pools that were turning over rapidly as a result of efficient N management, high soil microbial activity, and rapid plant N uptake. Composted yard waste inputs with relatively high C:N ratios in concert with limited use of labile organic fertilizers applied during peak plant N demand (e.g. ~5 kg N ha^-1^ as fish emulsion at 4–5 times in field 12) provided organic matter inputs with a range of N availability. A companion study showed how high potential activities of N-cycling soil enzymes (e.g. aspartase) but lower activities of C-cycling enzymes (e.g. β-glucosidase) in this set of fields reflect an abundant supply of C but N limitation for the microbial community, thus stimulating production of microbial enzymes to mineralize N [10]. Plant roots can effectively compete with microbes for this mineralized N, especially over time and when plant N demand is high [69–71]. High root expression of GS1 in these fields indicates that root N assimilation was elevated and thus actual plant N availability and uptake was higher than low inorganic N pools would suggest (see below).

Fields from group 2 demonstrated N surplus, showing similar yields to group 3 but with lower total and labile soil C and N and a higher potential for N losses, given much higher soil inorganic N (e.g. 16.2 vs. 2.9 μg NO_3_^-^ -N g^-1^ at anthesis in surface soil). While actual N losses depend on a host of factors (e.g. soil moisture, soil texture, irrigation), high soil NO_3_^-^ is considered an indicator for N loss potential [5,72,73]. Results from a companion study support the idea that soil microbes were C rather than N-limited in these fields, showing higher potential activities of C-cycling soil enzymes but low activities of N-cycling soil enzymes [10], the inverse of group 3 (see above). An alternative multivariate clustering approach based on an artificial neural network suggests multiple potential drivers of higher inorganic N pools in these fields, including both management factors and soil characteristics (S2 Fig). For instance, field 4 had strong indications of surplus N (e.g. the highest soil inorganic N of all fields, high N in tomato tissue, but also the highest yields) driven at least in part by a large application of seabird guano (~108 kg N ha^-1^), a readily-mineralizable organic N fertilizer, at tomato transplanting when plant N demand is low. In contrast, higher inorganic N in field 8 was likely driven by low plant N demand based on very low soil P availability (4.9 μg g^-1^ Olsen P) [10], which resulted in plant P limitation. These site-specific problems were identifiable due to the focus on variability across similar organic fields and illustrate the need for site-specific approaches to reduce N losses.

Finally, the two fields included in group 1 were exemplary of N deficiency, in which low N availability compromises crop productivity but also likely limits N losses within the growing season. While low soil NH_4_^+^ and NO_3_^-^ concentrations were similar to group 3, low total and labile soil organic matter and poorly-timed organic matter inputs (a single application of poultry and cow manure the fall prior to the growing season with no supplemental organic fertilizers) compromised microbial activity (e.g., the lowest potential soil enzyme activities [10]) and likely limited N mineralization.

### Root gene expression as a “plant’s eye view” of soil N cycling

Cytosolic glutamine synthetase GS1 encodes for the enzyme that catalyzes the addition of NH_4_^+^ to glutamate, the former resulting from either direct uptake of NH_4_^+^ from soil or reduction of NO_3_^-^ in roots. GS1 is thus the gateway for N assimilation in roots and is upregulated to increase root N assimilation capacity [32]. Similar levels of GS1 expression in groups 2 and 3, in spite of large differences in soil NH_4_^+^ and NO_3_^-^ concentrations at the anthesis sampling, suggests that plant N availability is indeed higher in group 3 fields than would be expected based on measurement of inorganic N pools alone. The low levels of GS1 expression found in fields with clear N deficiency (group 1) supports this idea. These results complement recent experimental approaches that showed rapidly increased expression of GS1 in tomato roots in response to a pulse of ^15^NH_4_^+^-N on an organic farm soil, which was linked to subsequent increases in root and shoot ^15^N content, even when this pulse did not significantly change soil inorganic N pools [30,31]. GS1 transcripts and glutamine synthetase enzyme activity also increased with increasing NH_4_^+^ and NO_3_^-^ availability in sorghum roots [74], suggesting this response may be widespread among plant species. Interestingly, inclusion of soil GWC in multiple linear regression models increased the proportion of GS1 expression variability explained to nearly 30% (S1 Fig); soil water content increases microbial activity as well as the mass flow and diffusion of inorganic N to roots [75]. Further research will undoubtedly show how other factors like crop physiological N demand relative to C fixation and P availability increase the interpretability of N uptake and assimilation gene expression in roots [28].

### Increasing multiple ecosystem functions in heterogeneous landscapes

The N cycling scenarios identified on this set of organic fields corresponded at least in part with landscape clusters based on landscape and soil characteristics (Table 2). Fields that balanced high yields with low potential for N loss and high internal N cycling capacity (group 3) were part of PAM cluster 1, which had the highest productive capacity rating (i.e. Storie Index). Landscape clusters encompassing more marginal soils (i.e. PAM clusters 3 and 5) included both low-yielding fields exhibiting N deficiency (group 1) or high-yielding fields that used inputs of highly available N like seabird guano to alleviate N deficiency (group 2). But these inputs led to the highest soil NO_3_^-^ levels and thus came at the cost of higher potential for N loss. Long-term efforts to increase internal soil N cycling capacity would help alleviate both N deficiency and the need for such large inputs of labile N. Whether farmers are willing to invest in management to increase soil N cycling capacity depends in part on how likely they perceive the benefits to be, especially on marginal soils [9]. The discussions that we had with each farmer in this study indicated genuine interest in adaptive management to further tighten plant-soil N cycling, but this may not always be the case. Indeed, the proportion of management vs. inherent soil characteristics responsible for driving differences in N cycling is challenging to untangle. Farmers may allocate more resources to more productive land and likewise fewer resources to more marginal land [76], or may selectively transition more marginal land to organic management [77]. Documenting the multiple services provided by increases in soil quality and facilitating information exchange among organic growers such as through the landscape approach used here may help build momentum for efforts to improve soil quality and plant-soil-microbe N cycling [78].

## Conclusions

A desirable scenario, i.e. tightly-coupled N cycling, with high crop yields, high soil N supplying capacity, and low potential for N losses, was found on organic farm fields with the highest total and labile soil C and N and management that provided a range of organic matter inputs. The heterogeneity across the 13 organic fields showed that reliance on typical metrics of N availability (e.g. soil NO_3_^-^) alone is insufficient for adaptive N management on organic farms. Low soil NO_3_^-^ may indicate either an N deficient system or tightly-coupled N cycling, so combining multiple indicators is essential. Root gene expression levels may eventually become a cost effective monitoring tool as technology becomes more accessible, particularly if multiple abiotic and biotic stressors could be assessed simultaneously. The landscape approach provided the range of variability necessary to evaluate indicators of N cycling and set the stage for adaptive management to speed progress toward the challenging goal of achieving multiple ecosystem services in intensively-managed, heterogeneous landscapes.

## Supporting Information

S1 Fig Relative expression of cytosolic glutamine synthetase GS1 in roots in relation to measures of C and N availability. Shown are residuals of GS1 after regression against gravimetric water content (GWC). The coefficient of determination given is from the full model (GWC + the soil variable), and the p-value given is the significance of the slope for the soil variable in the full model. Root samples were from field-grown Roma-type tomatoes across 13 organic fields in Yolo Co., California, USA. All soil measures were from surface soil (0–15 cm). The shaded region along the regression line is the 95% confidence interval for the mean of GS1.
(PDF)

S2 Fig Kohonen self-organizing map (KSOM) analysis of 24 plant, soil, and microbial variables from 13 organically-managed Roma-type tomato fields in Yolo Co., California, USA. a) Results of the KSOM analysis and color-coded legend showing variables included in the analysis (see Table 5). Wedges represent codebook vectors, i.e. the summary of variable values for each cell in the KSOM. The size of the color-coded wedges describes the relative magnitude of that variable for a given cell, i.e. large wedges represent large values and small wedges represent small values. Wedges that appear to be missing represent extremely low values of that variable relative to the rest of the dataset; there were no missing values in this analysis. Color families (e.g. red vs. green) correspond to related variables. Appended suffixes “t1”, “t2”, and “t3” refer to sampling time: pre-transplant, mid-season, and harvest, respectively. b) Position of field samples within each cluster. The position of the numbers within a cluster is not meaningful.
(PDF)

S1 Table Geographic coordinates of 13 organically-managed Roma-type tomato fields sampled over the 2011 growing season. (XLSX)

S2 Table Soil inorganic N pools across all fields, times, and depths. Data are presented as back-transformed means (n = 6) ± 95% confidence intervals.
(XLSX)

S3 Table Aboveground biomass and biomass N of organic Roma-type tomatoes. Data are from harvest sampling at the end of the 2011 growing season.
(XLSX)
