# Supplementary figures and images for: Tightly-Coupled Plant-Soil Nitrogen Cycling: Comparison of Organic Farms across an Agricultural Landscape
Source: PLoS One. 2015 Jun 29;10(6):e0131888. doi: 10.1371/journal.pone.0131888 (PMC4487741; doi:10.1371/journal.pone.0131888)

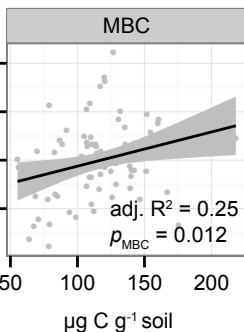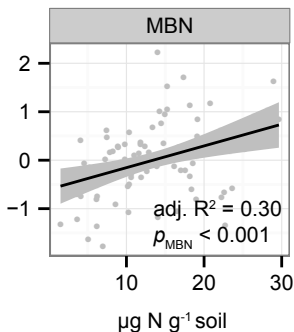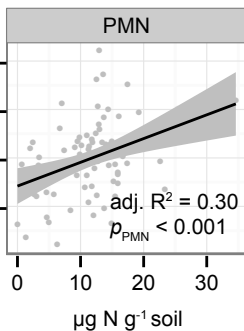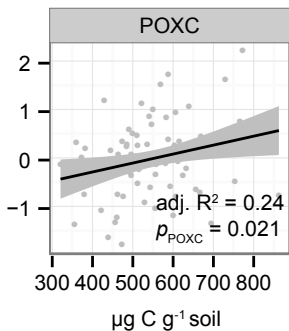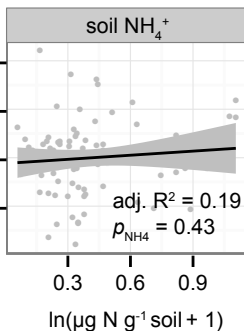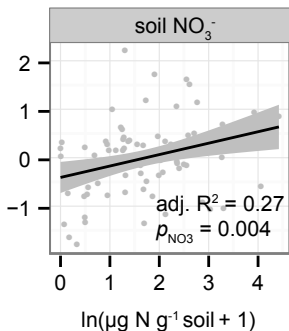

Supplement: S1 Fig — Shown are residuals of GS1 after regression against gravimetric water content (GWC). The coefficient of determination given is from the full model (GWC + the soil variable), and the p-value given is the significance of the slope for the soil variable in the full model. Root samples were from field-grown Roma-type tomatoes across 13 organic fields in Yolo Co., California, USA. All soil measures were from surface soil (0–15 cm). The shaded region along the regression line is the 95% confidence interval for the mean of GS1. (PDF) [file pone.0131888.s001.pdf]

(a)

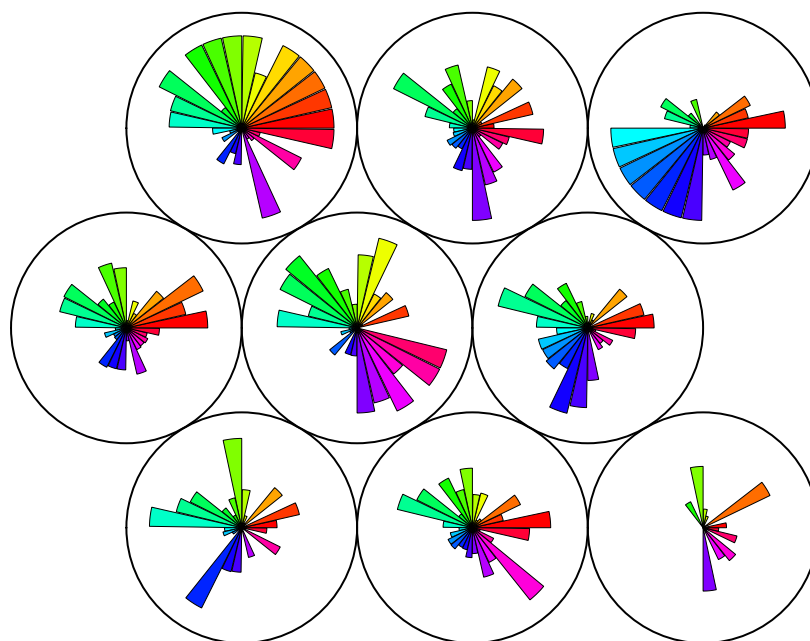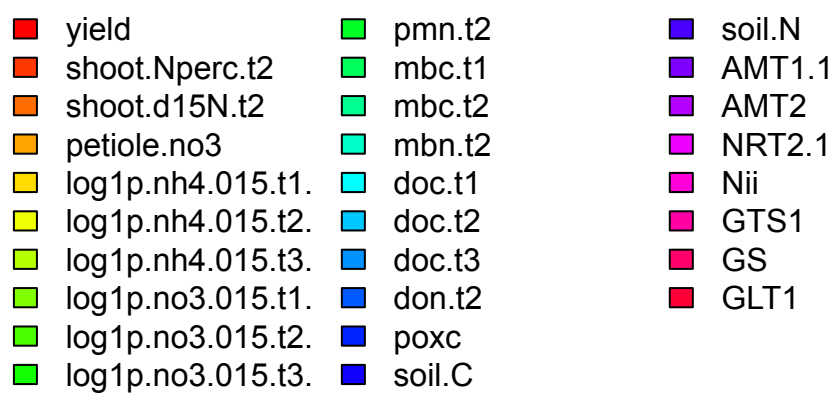

(b)

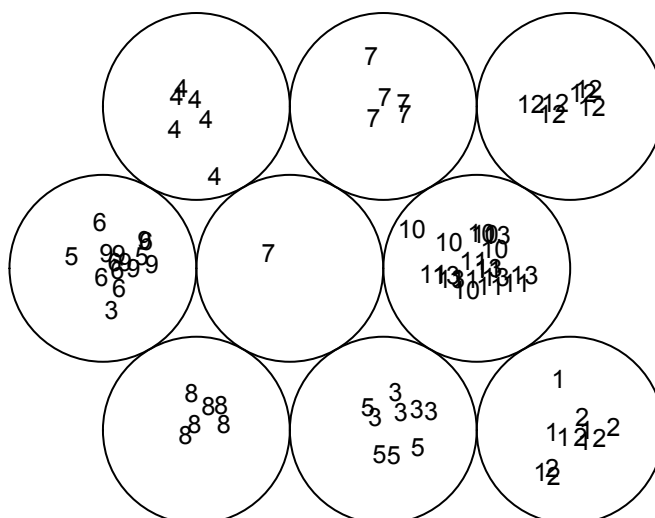

Supplement: S2 Fig — a) Results of the KSOM analysis and color-coded legend showing variables included in the analysis (see Table 5). Wedges represent codebook vectors, i.e. the summary of variable values for each cell in the KSOM. The size of the color-coded wedges describes the relative magnitude of that variable for a given cell, i.e. large wedges represent large values and small wedges represent small values. Wedges that appear to be missing represent extremely low values of that variable relative to the rest of the dataset; there were no missing values in this analysis. Color families (e.g. red vs. green) correspond to related variables. Appended suffixes “t1”, “t2”, and “t3” refer to sampling time: pre-transplant, mid-season, and harvest, respectively. b) Position of field samples within each cluster. The position of the numbers within a cluster is not meaningful. (PDF) [file pone.0131888.s002.pdf]
